# The BioPrompt-box: an ontology-based clustering tool for searching in biological databases

**DOI:** 10.1186/1471-2105-8-S1-S8

**Published:** 2007-03-08

**Authors:** Claudio Corsi, Paolo Ferragina, Roberto Marangoni

**Affiliations:** 1 Dipartimento di Informatica, University of Pisa, Italy

## Abstract

**Background:**

High-throughput molecular biology provides new data at an incredible rate, so that the increase in the size of biological databanks is enormous and very rapid. This scenario generates severe problems not only at indexing time, where suitable algorithmic techniques for data indexing and retrieval are required, but also at query time, since a user query may produce such a large set of results that their browsing and "understanding" becomes humanly impractical. This problem is well known to the Web community, where a new generation of Web search engines is being developed, like Vivisimo. These tools organize on-the-fly the results of a user query in a hierarchy of labeled folders that ease their browsing and knowledge extraction. We investigate this approach on biological data, and propose the so called The BioPrompt-boxsoftware system which deploys ontology-driven clustering strategies for making the searching process of biologists more efficient and effective.

**Results:**

The BioPrompt-box (Bpb) defines a *document *as a biological sequence plus its associated *meta-data *taken from the underneath databank – like references to ontologies or to external databanks, and plain texts as comments of researchers and (title, abstracts or even body of) papers. Bpboffers several tools to customize the search and the clustering process over its indexed documents. The user can search a set of keywords within a specific field of the document schema, or can execute Blastto find documents relative to homologue sequences. In both cases the search task returns a set of documents (*hits*) which constitute the answer to the user query. Since the number of hits may be large, Bpbclusters them into groups of homogenous content, organized as a *hierarchy of labeled clusters*. The user can actually choose among several ontology-based hierarchical clustering strategies, each offering a different "view" of the returned hits. Bpbcomputes these views by exploiting the meta-data present within the retrieved documents such as the references to Gene Ontology, the taxonomy lineage, the organism and the keywords. Of course, the approach is flexible enough to leave room for future additions of other meta-information. The ultimate goal of the clustering process is to provide the user with several different readings of the (maybe numerous) query results and show possible hidden correlations among them, thus improving their browsing and understanding.

**Conclusion:**

Bpb is a powerful search engine that makes it very easy to perform complex queries over the indexed databanks (currently only UNIPROT is considered). The ontology-based clustering approach is efficient and effective, and could thus be applied successfully to larger databanks, like GenBank or EMBL.

## Background

Biological databanks today offer a huge amount of information concerning DNA, proteins and other kinds of biological issues. Paradoxically, this sets a problem to biologists who want to discover as much information as possible about a specific topic. In the early 80's, when the first biological databank was built (e.g., PIR), the small amount of data available made it possible to browse all those related to a user query. Today the situation is quite different both in terms of size of the available biological data and the amount of meta-information associated to them – like molecular functions, biological processes or cellular components involved with genes or proteins. The risk is that biologists may miss the *relevant *information present in a databank when performing their searches because of the possibly large amount of results returned by a user query. This may force the biologist to manually, and thus impractically, scan those results or exploit tools – like GO [1] and NCBI Entrez [2] – in order to isolate the interesting entries for further and direct analysis.

This context reminds us of a similar field: the Web. Third-generation search engines are currently providing new tools to better and better satisfy the "user needs": personalization (e.g. Eurekster, see [3]), user behavior profiling (e.g. Google, Yahoo), query term suggestion (e.g. Ask), are just a few of them. Web-results clustering is another approach that has been recently introduced to help users in their difficult retrieval task. It has been pioneered by NorthernLight (1996) and then recently made famous by Vivisimo. The efficiency of the technology has been recognized with the "best meta-search engine award" from 2001 to 2003 by SeachEngineWatch. Last year, Dogpilewas voted as the most innovative IR-tool; Dogpile has been granted licence to Vivisimotechnology [4]. The basic idea is that the results returned by a (meta-)search engine are clustered into a hierarchy of folders which are labeled with intelligible sentences capturing the "theme" of the results contained in them. Users can therefore navigate through the folder hierarchy driven by the "need" behind their query. In this way users are not limited to look at the first ten results (more than 85% of web users do it), but they can immediately acquire several points of view on a larger set of them and thus narrow their search by clicking on some folders, or mine new knowledge by looking at the folder labels. These nice features have driven some authors to say that "clustering technology is the PageRank of the future", and several papers have been published on this subject (see [5-10]).

The Web shows some analogies and some obvious differences with the biological databanks mentioned before. The amount of data available, and usually returned by a (Web or Bio) search engine, is beyond any user ability to manually browse it. Also, any user has a "search need" which, especially in the biological context, is *informative *and thus aims at finding not only results matching the user query, but also at discovering relationships or properties among them. As far as differences are concerned, biological data are meta-tagged, structured and of high-quality since the meta-information comes from human experts; conversely the Web is unstructured, highly heterogeneous and its pages have usually poor quality. This makes the bio-search task potentially easier, or symmetrically, this should drive biologists to aim for more powerful search/mining tools.

In this paper we propose a new software system, called TheBioPrompt-box (Bpb), whose main goal is to pioneer the use of clustering technology on biological data in order to make the search task of biologists easier, more effective and more efficient.

## The BioPrompt-box: concepts and architecture

Even if Bpbis designed to manage any biological database, at present it has been restricted on UNIPROT only, because of two main reasons: UNIPROT has a relative small size, which makes it easier to be managed at a prototype level, like Bpbis; UNIPROT entries are supervised by humans, thus conferring them a high quality. Bpbdefines a document as a *structured list of fields*: one field contains an aminoacidic or nucleotidic sequence, all other fields contain the meta-data associated to this sequence accordingly to the underneath databank (currently, UNIPROT). These fields can be of two types:

1. *references *to data contained in others databanks (like Gene Ontology [1] or PubMed [11] to refer publications related to the sequence),

2. *texts *consisting, for example, of information about the organism and its taxonomy lineage, the submitters comments or the set of keywords characterizing the sequence itself.

To improve the recall of the search phase, every references to GO is also expanded into its *plain description *at indexing time. This means that each Bpbdocument will has both the *references *to GO both the *texts *(aka the descriptions) of the referred GO terms. This way the biologist can search for documents using terms featuring a family of GO elements instead of asking for a specific GO term. To achieve this result, at indexing time Bpbuse a local instance of GO. This is just one of the multiple way to improve the recall of the search phase. Others references (for instance the one versus PubMed) can be resolved at indexed time and the referred data can be associated to the document. These strategies can be easily added to the indexing process of Bpband may be part of a future release.

As an example of fields forming a Bpbdocument, we report the following:

• entry_sequence: the sequence (taken from UNIPROT)

• keyword_text: a keyword associated to the sequence

• protein_name: the name of the protein (valid for UNIPROT databank)

• organism_name_scientific: the scientific name of the organism

• organism_name_common: the common name of the organism [optional]

• entry_comment: a textual comment about this sequence

• GO_term: a reference to Gene Ontology

• organism_taxonomy: taxonomy lineage of the organism

• citation_title: the title of the cited paper

• document_superfield: the content of all other fields merged together. The goal is to allow an effective search simultaneously over all fields, by taking advantage of the indexing tool we adopt (see below).

Some of these fields can be present more than once in a document to allow multi-value content.

Bpb has been designed to be a scalable, full-featured and extensible search and clustering engine. It is written entirely in Java and structured as a typical 3-tier web application. As any other search engine, Bpbfeatures two main phases: indexing and querying. In the former phase, Bpbbuilds on disk the data structures used to subsequently support fast user searches over the indexed (structured) documents. As mentioned before, the meta-data present in the indexed documents consist of a dump of the UNIPROT databank (precisely, we indexed the UniProtKB databank available at EBI in January 2006). together with the descriptions of the referred GO terms resolved exploiting a local running version of the ontology (kept synchronized with the last official version). Periodical rebuilds are therefore needed to keep the Bpbindex up-to-date. The indexing task is performed by means of two well-known software libraries that guarantee efficiency and scalability: Apache Lucene [12], a high-performance and open-source text search engine, and Apache Commons Digester [13], a library for processing XML data (which is the format used to encode every structured document).

At query time the Bpbindex can be deployed to retrieve a *candidate *set of documents satisfying a user query. A query may be formulated in two ways: (1) as a set of keywords to be searched in any or all textual fields constituting the structured document (this is the typical *bag-of-words *paradigm of Web searches), or (2) as a specific protein sequence to be *matched for homology *using the BLAST algorithm over the field entry_sequence (this is the typical similarity-based search in bioinformatics). The resulting set of documents satisfying a user query is then *ranked *accordingly to a specific ranking function which depends on the query type. In the former case, Bpbuses the standard cosine similarity measure [14] as implemented by Apache Lucene, whereas in the latter case Bpbemploys the similarity score assigned by the instance of the BLAST algorithm provided by the Washington University (i.e. Wu-Blast[15]). The ranked list of results will be showed to the user, as it commonly occurs in any search engine.

Nonetheless Bpbis a clustering engine too. So that, once a query is resolved and its candidate set of results is determined and ranked, the clustering process comes into play. This phase of Bpbconsists of grouping the results accordingly to the homogenous content of some of their fields. The user can actually choose (via the GUI) among several ontology-based clustering strategies, each offering a different "view" on the returned results according to their associated meta-data such as: the references to Gene Ontology (i.e. field GO_term), the taxonomy lineage (i.e. field organism_taxonomy), the organism (i.e. the fields organism_name_scientific and organism_name_common), and the keywords (i.e. the field keyword_text). Of course, the approach is flexible enough to allow additions of other meta-information to the document schema. We point out that the clusters within a single view may *overlap*, because one result may satisfy different properties, and clusters may be organized into a *hierarchy*, in order to capture the *parent-child *relationships that might hold among pairs of them. As an example, one molecular function may be the specialization of another. Bpbthen visualizes for each cluster in the hierarchy a *string label *and the *cluster size*. The former information is intended to provide a succinct and meaningful description of the cluster content, while the latter provides an indication of the relevance of the cluster based on the number of items contained in it. Of course the more readable the cluster labels are, the more effective is the subsequent browsing and mining processes executed by the biologists on this *hierarchy of labeled folders*. It goes without saying that, differently from the indexing phase, the clustering phase must be executed on-the-fly at query time and must operate just on the document set returned by the user query. Consequently Bpbmust find a good compromise among detailed clustering (to represent at best the distinct properties characterizing the set of query results), readability of the cluster hierarchy (to help at best the biologists in their mining task), and efficiency of the clustering process (to avoid an unacceptable long waiting time by users).

The net result offered by a good hierarchy of labeled folders to the biologist is a compact way of *summarizing *all the results relative to the query. This provides the biologist with several different readings of the (maybe numerous) query results, their distributions over various ontology-based topics or organisms, and possible hidden correlations among them. Subsequently to their query and hierarchy reading, the biologists can either formulate a *new *query, by means of a more specific set of keywords, or they can *narrow *the current set of results by selecting just a few specific folders within the hierarchy. In both cases, the labels assigned to the folders help either in the generation of new knowledge for new-query formulation, or in the browsing process. We remark here that this approach to the browsing and understanding of query results is *orthogonal *to the classical linear-scan of the ranked list of hits, and shows its full power in the presence of *informative *or *under-specified *queries, as deeply illustrated for Web-search engines and as it is common in current biological databanks.

In its last step Bpboffers a twofold representation of the query results: (1) the classical *flat list *of results ranked according to some relevance-function, and (2) a hierarchy of labeled folders computed according to some clustering criteria selected by the user. Then the user can either scan through the flat-list, or can navigate the hierarchy and mine new knowledge from the cluster labels.

In the rest of this paper we will detail the search phase from the user's point of view and we will provide more insights on the algorithms underlying the clustering process.

### The search phase

**Bpb **offers various types of query operators, like the classical boolean operators (like AND, OR and NOT), or the more sophisticated *phrase searches *(by quoting the constituting terms), and *wild-cards *(by using the symbol *). Moreover, since the indexed documents are organized into *fields*, the user can issue *highly selective *or *general *queries. In the former case, the user specifies one or more (meta-)fields over which the query is matched; in the latter case, no field is specified by the user, and thus the search is performed over the document_superfield containing the content of all the others fields, achieving in this way a fast search over all the document fields simultaneously. As an example of selective query, the user can issue: glutamate* AND organism_taxonomy:mammalia, to look for documents about the *glutamate *and relative to the *mammalian organism*.

Another interesting type of search supported by Bpbis the one related to the meta-data extracted from *Gene Ontology *(GO), and attached to every document in the indexed data collection (see the previous section). Each document may indeed have references to GO terms that describe the molecular functions and the biological processes involved with the corresponding databank entry, and may contain information about where these functions or processes take place (i.e. cellular compartments). By exploiting the indexed information, the users can boost the precision of their searches by narrowing the query to a specific function, process or component without specifying exact terms in GO, but just describing them via the Web-classical "bag-of-words" search paradigm. For example, the user can look in Bpbfor all the proteins related to a specific biological process described by the GO term [GO:0005216] named "ion channel activity" by just issuing a phrase search with these three keywords. We believe that this feature is helpful to anyone looking for sequences with a specific function, process or cellular component but doesn't know the exact GO terms that identify them. Currently, the same type of query could be answered by issuing multiple searches in sequence and ontology databanks like Go: however these combined searches would usually require more than one attempt to produce interesting results. In fact, the phrase query above does not produce results on UNIPROT (we are referring to the search function provided at [16] over the SwissProt collection), whereas it produces 37 results on Bpb. If we remove the quotes, UNIPROT returns 98 results. Of course, Bpbsupports searches for GO terms, as well.

As mentioned in the previous section, a user can issue two types of queries: index-based and BLAST-based. At any time of the search process, the user can issue a BLAST-based search on a subset of sequence results currently returned by Bpb. The selection and running options are offered through the GUI.

Independently of the type of issued query, Bpbproduces a list of documents which answer the query posed by the user. If the list is small enough to be manually scanned, the user should do it. Otherwise, it is more effective for the biologist to exploit the hierarchy of labeled clusters produced by Bpb.

### The clustering process

Clustering deeply exploits the meta-data constituting the documents indexed by Bpb.  Bpbmay produce various kinds of labeled clusters which depend on the specific data deployed in the aggregation and labeling process. This brings to the concept of "view" previously introduced in this paper. Three points relative to the clustering process should be addressed:

1. How to perform the clustering;

2. Which heuristics apply in order to produce few but significant clusters;

3. How to use the clusters to improve the precision and the recall of the user search.

The goal of the clustering process is to group the documents returned as query results into homogeneous groups (i.e. clusters) according to a given property which is chosen by the user at query time via the GUI. For instance, a user can choose to group together documents with the same keywords in the keyword_text field, or with the same GO terms in the GO_term field. Currently, the views supported by Bpbare the following:

#### Taxonomy view

The clusters are generated on the base of the taxonomic information present in the field organism_taxonomy of the retrieved documents, which contains the entire taxonomic path from the kingdom to the species of the relative organism. This view creates a *reduced form *of taxonomic tree in which the taxonomic nodes are the labeled clusters only, and the parent-child relationships are derived from the taxonomic information "as-is" given by the NCBI taxonomy (since we indexed UNIPROT). Other databanks could be adopted without changing the current Bpbarchitecture. Of course, the reduced taxonomic tree may present many unary paths.  Bpbthus performs a "compression" of those paths by removing all the intermediate unary nodes (clusters). The ratio underlying this approach is that the removed nodes (clusters) do not provide useful information to the user who wants to see the distribution of the results among the different taxonomy clusters. Moreover, these unary nodes would unreasonably enlarge the user time for the hierarchy browsing.

#### Keyword view

This view is based on the content of the keyword_text field that textually describes (without the use of an ontology) each retrieved document via a list of terms (keywords). This is different from the GO Terms view below, since the GO terms belong to a strictly controlled vocabulary, while those keywords are manually inserted in the data file by the submitters and, even if they are always concerned with the biological properties of the sequence, they might be chosen from common language. However, since Bpbcurrently works on the UNIPROT databank, the used keywords are from a predetermined vocabulary. Again, the architecture of Bpbis flexible enough to host any set of keywords in the field keyword_text, as future releases of the software might offer.

#### Organism view

Each document is related to a specific organism indicated in the document itself in the fields organism_name_common and organism_name_scientific with a content like Homo Sapiens or Mus Musculus. For each organism cited in the retrieved documents, Bpbbuilds a cluster labeled with the organism name and containing all documents corresponding to that organism. These clusters can be considered as the leaves of the taxonomy tree, so no hierarchy is built in this view. The organism view allows the biologist to focus his search process on some specific organisms of interest.

#### Go Terms view

For each GO terms cited in the retrieved documents set, we group together all the hits that refer to that term, and label the formed cluster with the term itself. Then we discover parent-child relationships by querying GO to find whether two terms are related. This actually means that a path between them does exist in the Gene Ontology (the use of a local instance of GO, instead of querying over the Internet some web service providing access to the ontology, makes fast this process). Given this information, Bpbis able to organize the cited terms (and thus their clusters) in a more sophisticated data organization, namely a Directed Acyclic Graph (as GO does).

#### GO Upward Paths view

This is the most sophisticated clustering approach offered by Bpb. GO is a DAG whose nodes are labeled with the name of a molecular function, biological process or cellular component; and whose arcs model two kinds of relationships: *is_a *and *part_of*. In principle every protein of UNIPROT (our supported databank) is tagged with all the references to related terms in this ontology; however, the manual tagging process is still in progress [17]. Nevertheless, the quality of the linking and tagging information available in this DAG makes it an effective knowledge base for clustering and labeling the documents returned as a result of a user query. It is in fact reasonable to cluster together documents of the result set which share the same reference to GO (because of a common molecular function or biological process), or are "close" in the GO graph. We follow this idea by creating one cluster per GO term referenced by the query results, and put into this cluster the documents that reference that term (hereafter we will use term "documents" meaning the documents of the answer set). After that, we start from these terms (clusters) and visit backward the GO graph for a fixed number of steps (here we heuristically use 3 steps, which keeps the view meaningful and compact). For each visited term we form a cluster, labeled with that term, and containing the documents that refer to that term directly (if any), and all documents referring to terms that descend from it. For instance, suppose that we start from the term [GO:0005216] (ion channel activity). We create a cluster labeled with the term name and containing all the documents referring to this term directly. Going backward in the ontology we find two fathers for this term: [GO:0015268] (alpha-type channel activity) and [GO:0015075] (ion transporter activity). For these two new terms we create two clusters labeled with their names and containing all the documents that directly refer to these terms together with the documents contained in the descendant clusters, in this example only the one for [GO:0005216]. The created clusters will be presented to the user organized hierarchically as they appear in the ontology. In our example, the clusters for [GO:0015268] and [GO:0015075] will be presented at a higher level in respect to that of the cluster for [GO:0005216] which will be shown as a child of both.

This backward visit will eventually produce a forest of DAGs, since two starting terms may have a common ancestor at some level (≤ 3) and thus bring their connected components to merge, or may remain disconnected. Going upward in the GO ontology means that we are moving toward more generic and comprehensive concepts that can be then used to produce coarser and coarser clusters. Therefore, the shallowest terms will be related to generic concepts while the deepest terms will be related to more specific concepts. The GUI will display to the user the shallowest terms, and their clusters, which contain many documents because they represent more generic concepts. Starting from these clusters, the user can browse the deepest clusters containing a few more-specialized documents. We note that the upward visit (up to three steps) introduces new terms which are nonetheless "related" to the ones referred to, by the retrieved documents.

A comment is in order at this point. In principle, the clustering process should take place over the entire set of results produced by a user query. However this approach can be slow, because of the sophisticated clustering strategies adopted, and might jeopardize the precision of the clustering hierarchy, because of the presence of many clusters and maybe outliers. In order to overcome these problems, Bpbrestricts the clustering process to only the *top-n *ranked documents of the result set, thus mimicking Vivisimo. This algorithmic choice assumes that the ranking function takes care of selecting the most interesting and representative results among the ones returned by Bpbfor the user query. The value of *n *is crucial, it is currently set to 100, but it may be obviously changed according to the user needs.

It is important to note that these four views are not *mutually exclusive*, in the sense that at any time of the searching process the user can switch from one view to the other by just clicking on the proper button on the GUI. This flexibility allows the users to interact with the current set of results in the most effective way, by choosing the view that best refines the current query. The *refinement *may be performed by either clicking on a cluster label, or by performing a *multiple selection *over various clusters, possibly residing at different levels of the cluster hierarchy. In the former case, Bpbautomatically composes and executes a new query by combining in AND the current query with the selected cluster labels. In the latter case, the current result set is narrowed by keeping only the results that belong to the selected clusters.

## Results and discussion

**Bpb **has a very simple start page (in the "Google-like" philosophy) which accepts the query string, as either a keyword-based query or as a sequence to be matched via the BLAST algorithm. The page of results consists of two frames, the one on the right shows the flat-list of ranked results, the other on the left shows the hierarchy of labeled folders as computed according to the chosen view (GO Upward Path is the default).

**Figure 1:**
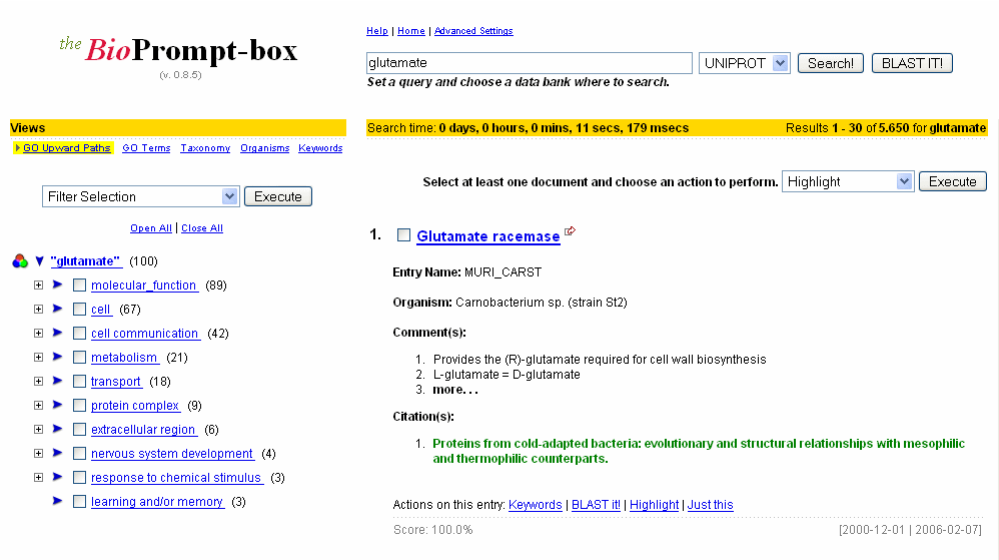
**Screenshot of Bpb results page**. A screenshot of **Bpb **after the query "glutamate". On the top, the query box with the options menu; on the left, the different views the user can navigate in; on the right, the flat and ranked list of results.

**Figure 2:**
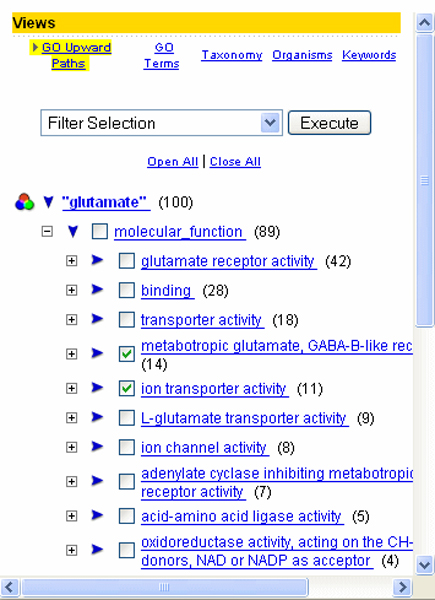
**Selecting clusters on one view**. This figure details the "Go Upward Paths" view for the query of Fig. 1. Here the user can select specific clusters to refine her search.

**Figure 3:**
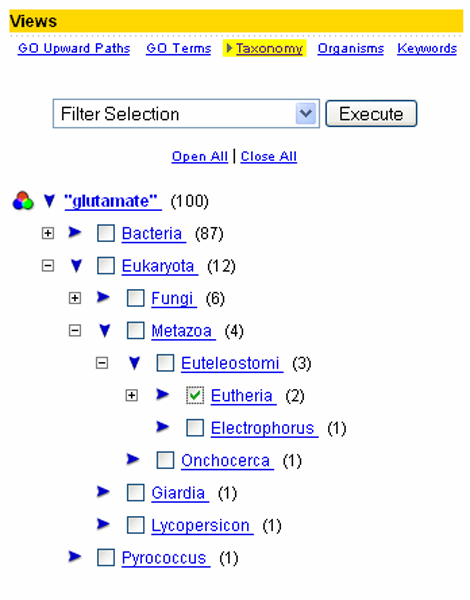
**Selecting clusters on another view**. This figure shows how a user can select another view (in this case: Taxonomy) and select clusters in it. The usage of different views, and the possibility to restrict the search within specific clusters in this view, make it very easy to compose complex queries in **Bpb**.

Figure 1 shows a snapshot of Bpbafter the query "glutamate". Figure 2 shows how a user can select different clusters from the Go Upward Path view in order to narrow the search on them. The GUI allows the user to change the view and automatically highlight the documents of the clusters which were selected in the previous one. This allows the user to keep track of changes in the views. Figure 3 shows how a user can select different clusters under different views, in order to perform restrictions of the query on the specified clusters only. Using this feature, the user can set very complex queries in a very easy way: for example, one can search for a molecule playing a generic biological function (e.g. "glutamate receptor"), expressed in one only taxonomic class (e.g. "Eutheria") and sharing some keywords with other molecules. Perhaps, this is the most important and useful feature of Bpb. Indeed, queries both too generic as well as too specific, are not so useful because they always produce either a too large or a too small number of results. On the contrary, queries having an intermediate specificity generate a relatively large set of results (more than 5600 for the query "glutamate"), and in this case, the multi-view approach of Bpbis very useful in immediately focusing the query on the subset of interest. These kinds of queries are, in practice, the most interesting for biologists.

Bpb is not the only search and clustering engine in the literature acting over many ontologies (i.e. GO Upward Path, GO Terms, Organism Taxonomy, Keywords, etc.). Other projects, like Clustermed [18], are exploiting the clustering approach but over only bibliographic databases, like PubMed [11]. Bpbdiffers from them in two main respects: (1) it uses several kinds of meta-data associated to the biological sequences (currently taken from UNIPROT) to perform the search and the clustering process; (2) it offers the possibility to link ontology classification and homology searches, via the combination of clustering and BLAST-based searches.

## Conclusion

We have presented a powerful and versatile prototype of search and clustering engine for biological data, called Bpb.  Bpbindexes protein sequences and their meta-data extracted from UNIPROT databank. The user can issue keyword-based or BLAST-based searches over these indexed data thus retrieving a set of documents which are ranked according to proper relevance-functions. These query hits are then clustered by Bpbinto groups of homogenous content, organized as a *hierarchy of labeled clusters*. The user can actually choose among several ontology-based hierarchical clustering strategies, each offering a different "view" on the returned hits and each based on a different meta-data associated with the hits. The ultimate goal of Bpbis to provide the user with several different readings of the (maybe numerous) query results and show possible hidden correlations among them, thus improving the user browsing and understanding. Finally, the biologist can interact with the hierarchy in order to either formulate a new query or to narrow the current set of results by selecting just a few specific folders.

We remind the reader that the current version of Bpbsupports searches only on the meta-data drawn from the UNIPROT databank. Future releases of the software will support other interesting databanks, like GenBank and/or EMBL, will allow the user to process and cluster efficiently a larger set of top-*n *documents, and will support other ranking functions.

## Availability and requirements

• Project name: The BioPromt-box

• Project home page: 

• Operating system(s): Platform independent

• Programming language: Java

• Other requirements: Java 1.4.0 or higher, Tomcat 5.0 or higher

• License: free

• Any restrictions to use by non-academics: no

## Authors' contributions

The Authors have equally contributed to this work.
